# Enhancing Emergency Medicine Training Through Trauma-Informed Pelvic Exam Education

**DOI:** 10.7759/cureus.97363

**Published:** 2025-11-20

**Authors:** Ellen Dowling, Amrita Vempati, Alicia Willey, Ibis Rojas

**Affiliations:** 1 Emergency Medicine, Valleywise Health Medical Center, Phoenix, USA; 2 Emergency Medicine, Creighton University School of Medicine, Phoenix, USA; 3 Obstetrics and Gynecology, Valleywise Health Medical Center, Phoenix, USA; 4 Obstetrics and Gynecology, Creighton University School of Medicine, Phoenix, USA

**Keywords:** medical resident education, pelvic exam, refugee care, teaching in emergency medicine, trauma informed care

## Abstract

Objectives

Refugee and immigrant women are at high risk for gender-based violence (GBV) and frequently present to the emergency department (ED) as their first interaction with the healthcare system. Trauma-informed care (TIC) is essential to providing sensitive, safe care to these populations, particularly during pelvic examinations. Despite this need, formal TIC training for emergency medicine (EM) residents remains limited. This study evaluated the effectiveness of a TIC-focused workshop aimed at improving confidence and competence in performing pelvic exams on patients with a history of GBV.

Methods

A single-session workshop was delivered to 34 learners (EM residents and senior medical students) at an urban training site. The session included a didactic overview of TIC principles, followed by role-play scenarios and hands-on pelvic exam skills practice. Participants completed pre- and post-session surveys assessing familiarity, confidence, and anticipated use of TIC maneuvers and language. Descriptive statistics were analyzed using Microsoft Excel (Microsoft Corporation, Redmond, Washington).

Results

All 34 participants (11 PGY-1, 11 PGY-2, 5 PGY-3, 7 MS4) completed both surveys. Prior to the session, the majority reported being only slightly familiar with TIC (53%) and trauma-sensitive exam components (59%). Most (76.5%) reported low confidence in using trauma-informed maneuvers. Only 9% reported always using such techniques during pelvic exams. Following the workshop, confidence markedly improved: 65% felt somewhat confident, 24% moderately confident, and 9% extremely confident in using trauma-informed maneuvers and language. The session was rated as excellent or very good by 85-88% of participants. Nearly all (91%) indicated they would always or often apply the skills in future clinical practice.

Conclusion

A brief, focused workshop significantly improved EM learners' confidence and intent to apply trauma-informed approaches during pelvic exams. Scaling similar training across EM residencies may enhance patient safety, reduce re-traumatization, and promote culturally responsive, compassionate care. Continued integration of TIC into EM education is essential to improving outcomes for trauma-affected populations.

## Introduction

Background

Gender-based violence (GBV) is defined as "any harmful act that is perpetrated against a person’s will and that is based on socially ascribed (gender) differences between males and females" [1]. Refugees and displaced women are among the most vulnerable populations to GBV. Approximately one in three women globally experiences physical or sexual violence, with young women and girls being particularly at risk among refugees. Studies show that over 60% of displaced women in low- and middle-income countries experience GBV. Additionally, approximately 48.2% of refugees and internally displaced women in Africa have faced GBV. Prevalence rates for GBV among refugee women in Africa range from 31% to 80.6%, while internally displaced women experience rates ranging from 18.5% to 54.4% [2]. In specific populations, such as women from Ethiopia, Sudan, and Somalia, studies have shown that up to 70% have experienced GBV, often perpetrated by intimate partners or family members [3,4]. Among Malaysian refugees, the percentage is as high as 60% [5]. The percentage of GBV among refugees from countries such as Tanzania, Kenya, Iraq, Iran, Afghanistan, and the Democratic Republic of Congo is up to 80%, which significantly increased after the COVID-19 pandemic [6]. The prevalence of violence is not limited to conflict zones but extends to post-conflict regions, including refugee camps, transit areas, and host communities. Survivors often face a range of consequences, including poor pregnancy outcomes, maternal mortality, sexually transmitted diseases, stigma, loneliness, stress, and forced pregnancies. Mental health issues such as suicidal thoughts, anxiety, depression, and post-traumatic stress disorder (PTSD) are also common. Additionally, physical health consequences include injuries, chronic pain, gastrointestinal issues, and disabilities [2,7]. Furthermore, studies show that GBV has been associated with stillbirths, miscarriages, lower birth weights, and preterm labor [8,9].

In Phoenix, over 108,000 refugees have been resettled from 66 unique countries, including Sudan, Somalia, Nigeria, Rwanda, the Democratic Republic of Congo, and Ethiopia [10]. Our urban county hospital, located in the heart of Phoenix, serves as a safety-net institution for the community. Our emergency department (ED) sees approximately 1,500 refugee patients per year, many of whom are women suffering from GBV. Refugee and immigrant populations are more likely to experience GBV, and often, the ED is their first point of contact with the healthcare system. Importantly, studies of asylum-seeking women in the U.S. reveal that many survivors have endured lifelong exposure to multiple forms of violence, often from multiple perpetrators, rather than a single event [11]. This cumulative and complex trauma highlights the imperative for provider awareness, sensitivity, and structural support when caring for such patients [11].

Asylum seekers and refugee populations frequently present to the ED with both acute medical needs and complex trauma histories. Emergency physicians are uniquely positioned to recognize these histories and provide trauma-sensitive care. However, U.S. physicians often report limited training in trauma-informed care (TIC) and face numerous barriers to implementation, including time constraints, provider burnout, and lack of institutional support [12,13].

TIC principles were developed to guide healthcare providers in caring for patients who have experienced significant trauma. The six key principles of TIC include safety, trust and transparency, peer support, collaboration and mutuality, and the empowerment of voice and choice, alongside humility and responsiveness [14]. These principles are particularly important for providing sensitive care to women who have experienced GBV [14]. Studies show that using these principles can promote wellness and recovery among vulnerable populations [15].

Greenwald et al. propose practical changes to ED environments and workflows that can support trauma-informed practices despite the high-acuity nature of emergency care [16]. Despite the significant need, there are limited data on TIC education for physicians, especially within emergency medicine (EM), regarding pelvic examinations and how to care for patients with histories of GBV presenting with genitourinary complaints.

Importance

Refugee and immigrant women are disproportionately affected by GBV and frequently seek care in the ED, making it the first point of contact with the healthcare system for many. TIC is essential to mitigate re-traumatization and provide sensitive, effective care to these populations. Appropriate language and physical examination maneuvers are critical to ensuring a trauma-sensitive approach, especially for patients presenting with genitourinary concerns. EM residents must be trained in TIC principles to deliver this care effectively.

Goals of this investigation

The primary goal of this investigation was to evaluate the effectiveness of a training workshop designed to teach EM residents the six principles of TIC while performing pelvic examinations in the ED. Through this workshop, we aimed to enhance the residents' understanding of how to incorporate TIC principles into clinical practice when caring for refugee and immigrant women who may have experienced GBV. Furthermore, we sought to assess how helpful the training was in preparing residents to apply these principles in future clinical encounters with vulnerable patients.

## Materials and methods

Study design

This study was conducted at our urban EM training site, which consists of a three-year EM residency program. Each year has 16 residents, with a total of 48 residents across all years. The study took place during the weekly conference (January 17, 2024), which typically includes 25-30 post-graduate year (PGY)-1, PGY-2, and PGY-3 residents, 5-10 fourth-year medical students (MS), and various attending physicians. All learners present at the educational conference were invited to participate in the study. A total of 34 learners participated, comprising EM residents at various training levels and medical students.

Setting and subjects

The training session was led by the Medical Director of the Women’s Refugee Health Clinic at our institution, who is also an Obstetrician-Gynecologist (OB/GYN), and residents experienced in TIC pelvic exams. The training session was held in the hospital conference room during a scheduled educational session. All participants present were invited to complete a pre-session survey before the workshop and a post-session survey following the completion of the workshop.

Interventions

The training session was a single, one-hour workshop conducted during a scheduled EM conference. All learners who attended the conference were included as participants. The session began with a 20-minute Microsoft PowerPoint® presentation introducing the core principles of TIC, including safety, trust and transparency, peer support, collaboration and mutuality, empowerment of voice and choice, and cultural humility and responsiveness.

The didactic content also reviewed the "Three E’s" model of trauma (event, experience, and effect) and practical communication strategies for conducting trauma-sensitive physical exams. Learners discussed the importance of obtaining consent, explaining each step of an exam, and using neutral language, for example, using "exam table" instead of "bed" or "allow knees to fall to the side" instead of "spread your legs." Three case scenarios were presented to illustrate how TIC principles apply in common ED encounters, including care for patients who use substances, survivors of GBV, and distressed learners.

Following the presentation, learners participated in 40 minutes of hands-on practice using pelvic examination task trainers. This segment included role-playing exercises to reinforce trauma-sensitive communication and appropriate terminology, as well as guided demonstrations by OB/GYN physician facilitators on applying TIC principles during pelvic exams. Small-group sessions ensured that each participant had the opportunity to observe, practice, and receive feedback on examination techniques in a supportive learning environment. The session concluded with a brief group reflection on system-level barriers to implementing TIC and strategies to promote trauma-informed practice in emergency care settings.

Interventions and measures

To assess the effectiveness of the workshop, data were collected via pre- and post-session self-assessments administered on paper forms. The questionnaires were locally developed to reflect the workshop’s learning objectives and trauma-informed care principles, though they have not undergone formal validation. The pre-session survey included the following questions (Table 1).

**Table 1:** Trauma-informed care pre-session survey.

Survey Questions				
1. How familiar are you with trauma-informed care?				
Not at all familiar	Slightly familiar	Moderately familiar	Very familiar	Extremely familiar
2. How confident do you feel utilizing trauma-informed care in your practice currently?				
Not at all confident	Slightly confident	Moderately confident	Somewhat confident	Extremely confident
3. How familiar are you with the key components of a physical exam that is sensitive to patients who have experienced trauma?				
Not at all familiar	Slightly familiar	Moderately familiar	Very familiar	Extremely familiar
4. How confident do you feel using maneuvers during the physical exam that may help patients who have experienced trauma feel more comfortable and safe?				
Not at all confident	Slightly confident	Moderately confident	Somewhat confident	Extremely confident
5. How confident do you feel using language during the physical exam that may help patients who have experienced trauma feel more comfortable and safe?				
Not at all confident	Slightly confident	Moderately confident	Somewhat confident	Extremely confident
6. How regularly do you use trauma-informed language with patients?				
Never	Rarely	Sometimes	Often	Always
7. How regularly do you use trauma-informed maneuvers when performing a pelvic exam?				
Never	Rarely	Sometimes	Often	Always

Following the training session, participants completed a post-session survey, which included the following questions (Table 2).

**Table 2:** Trauma-informed care post-session survey.

Survey Questions				
1. How confident do you feel using maneurvers during the physical exam that may help patients who have experienced trauma feel more comfortable and safe?				
Not at all familiar	Slightly familiar	Moderately familiar	Very familiar	Extremely familiar
2. How confident do you feel using language during the physical exam that may help patients who have experienced trauma feel more comfortable and safe?				
Not at all confident	Slightly confident	Moderately confident	Somewhat confident	Extremely confident
3. How effective was the presentation in defining a "trauma informed approach to a physical exam"				
Poor	Fair	Good	Very good	Excellent
4. How effective was the presentation in teaching trauma-informed language and maneuvers during the physical exam?				
Poor	Fair	Good	Very good	Excellent
5. How useful was time available to practice trauma-informed language?				
Poor	Fair	Good	Very good	Excellent
6. How often will you use the content from this presentation in your clinical practice?				
Never	Rarely	Sometimes	Often	Always

The pre- and post-session survey questions were developed by the study team based on a review of published literature on TIC and trauma-sensitive physical examination, as well as the authors’ clinical and educational experience in EM and simulation-based training. Items were designed to assess familiarity, confidence, and self-reported practice behaviors related to TIC. The instrument was reviewed by the instructional faculty for clarity and face validity. The completed surveys were collected, and responses were entered into a Microsoft Excel spreadsheet (Microsoft Corporation, Redmond, Washington) for analysis.

Data analysis

Data were analyzed by comparing pre-session and post-session responses to assess any changes in familiarity, confidence, and the perceived effectiveness of the training. Descriptive statistics were used to summarize the responses, and comparisons were made to evaluate the impact of the training session on the participants’ knowledge and confidence in applying TIC principles, specifically during pelvic examinations.

## Results

A total of 34 participants completed the pre-session survey, consisting of 11 PGY-1 residents, 11 PGY-2 residents, 5 PGY-3 residents, and 7 MS4 students. The survey aimed to assess their familiarity and confidence with TIC practices and their use of trauma-sensitive techniques during physical exams.

Pre-session survey results

**Figure 1:**
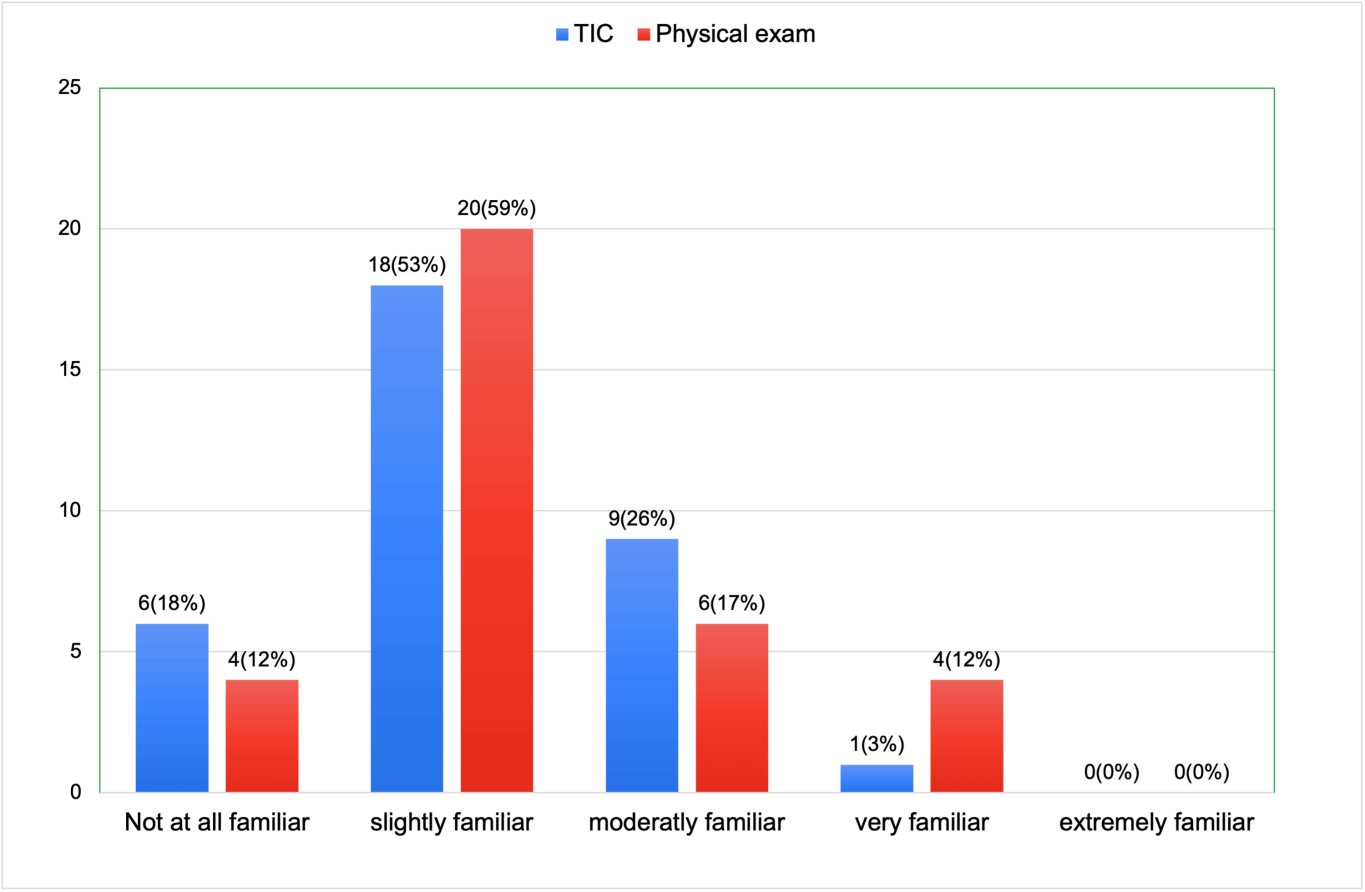
Pre-session survey results (a total of 34 respondents): how familiar are you with trauma-informed care and key components of physical exam that is sensitive to patient who experienced trauma? TIC: trauma-informed care.

The pre-session survey inquired about participants' familiarity with TIC. Eighteen of 34 respondents (53%) reported being slightly familiar with TIC, followed by nine moderately familiar (26%), six not at all familiar (18%), and one very familiar (3%). None of the respondents reported being extremely familiar. When asked about their familiarity with the key components of a trauma-sensitive physical exam, 20 participants (59%) reported being slightly familiar, six (18%) were moderately familiar, four (12%) were not at all familiar, and four (12%) were very familiar. None of the participants indicated extreme familiarity (Figure 1).

**Figure 2:**
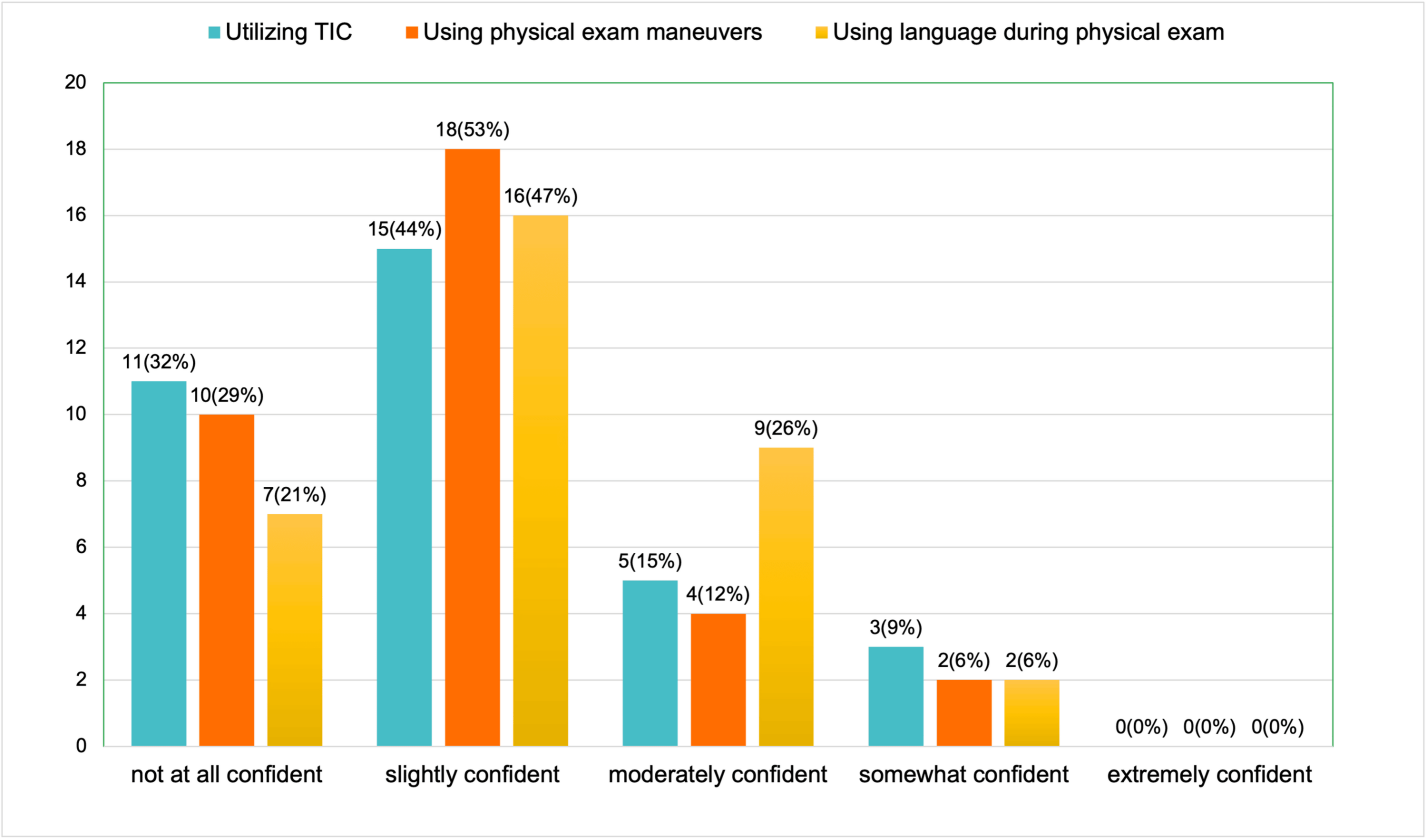
Pre-session survey results (a total of 34 respondents): how confident do you feel with utilizing TIC, using physical exam maneuvers and language during physical exam? TIC: trauma-informed care.

In terms of confidence in applying TIC (Figure 2), 11 participants reported feeling not at all confident (32%), 15 were slightly confident (44%), 5 were moderately confident (15%), and 3 were somewhat confident (9%). Notably, no participants indicated that they were extremely confident. Regarding confidence in using trauma-informed maneuvers during a physical exam to make patients feel more comfortable and safe, 10 respondents (29%) felt not at all confident, 18 (53%) were slightly confident, 4 (12%) were moderately confident, and 2 (6%) were somewhat confident. No participants reported feeling extremely confident. On the topic of using trauma-informed language during physical exams, 7 respondents (21%) were not at all confident, 16 (47%) were slightly confident, 9 (26%) were moderately confident, and 2 (6%) were somewhat confident.

**Figure 3:**
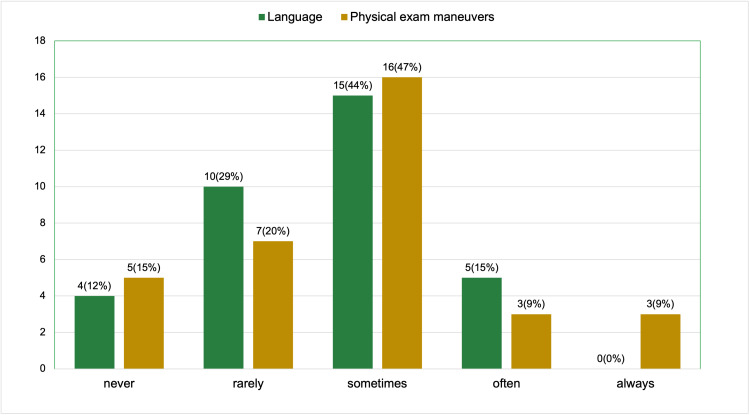
Pre-session survey results (a total of 34 respondents): how regularly do you use trauma-informed language and maneuvers during pelvic exam?

Regarding the regular use of trauma-informed language, 4 participants (12%) reported never using it, 10 (29%) rarely used it, 15 (44%) used it sometimes, and 5 (15%) used it often. No respondents reported always using trauma-informed language. The majority of participants (16 out of 34, 47%) reported using trauma-informed maneuvers during pelvic exams sometimes, followed by 7 (20%) rarely, 5 (15%) never, and 3 (9%) often. Notably, 3 participants (9%) reported always using trauma-informed maneuvers during pelvic exams (Figure 3).

Post-session survey results

**Figure 4:**
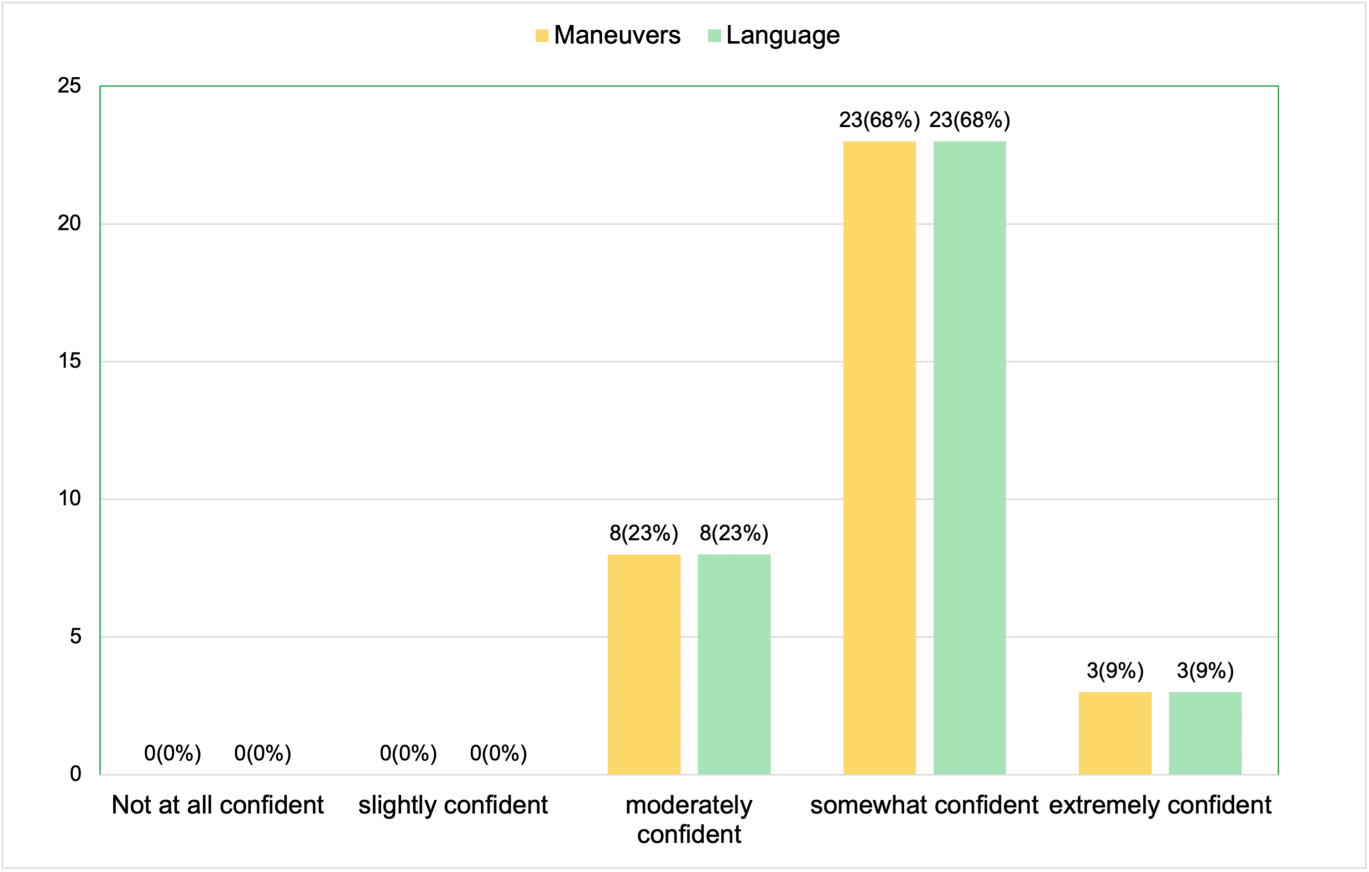
Post-session survey results (a total of 34 respondents): how confident do you feel using language and maneuvers during physical exam that may help patients who have experienced trauma?

The post-session survey results showed significant improvements in participants' confidence and perceptions regarding TIC after the session. When asked about their confidence in using trauma-informed maneuvers during the physical exam to make patients feel more comfortable and safe, 23 participants (68%) felt somewhat confident, 8 (23%) were moderately confident, and 3 (9%) were extremely confident. No participants reported feeling not at all confident or slightly confident. In terms of using trauma-informed language during physical exams, 23 participants (68%) felt somewhat confident, 8 (23%) were moderately confident, and 3 (9%) were extremely confident, with no participants indicating a lack of confidence (Figure 4).

**Figure 5:**
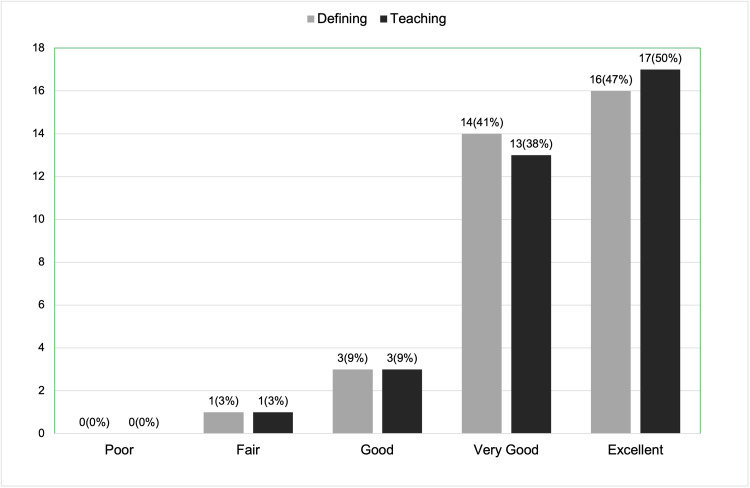
Post-session survey results (a total of 34 respondents): effectiveness of presentation in defining and teaching TIC physical exam skills. TIC: trauma-informed care.

Regarding the effectiveness of the presentation in defining a "trauma-informed approach to a physical exam," 16 participants (47%) rated it as excellent, 14 (41%) rated it as very good, and 3 (9%) rated it as good. For the effectiveness of teaching trauma-informed language and maneuvers during the physical exam, 17 participants (50%) rated it as excellent, 13 (38%) rated it as very good, and 3 (9%) rated it as good (Figure 5). 

When asked about the usefulness of the time available to practice trauma-informed language, 15 participants (44%) rated it as excellent, 13 (38%) rated it as very good, and 6 (18%) rated it as good. Notably, no participants rated the time as poor or fair.

**Figure 6:**
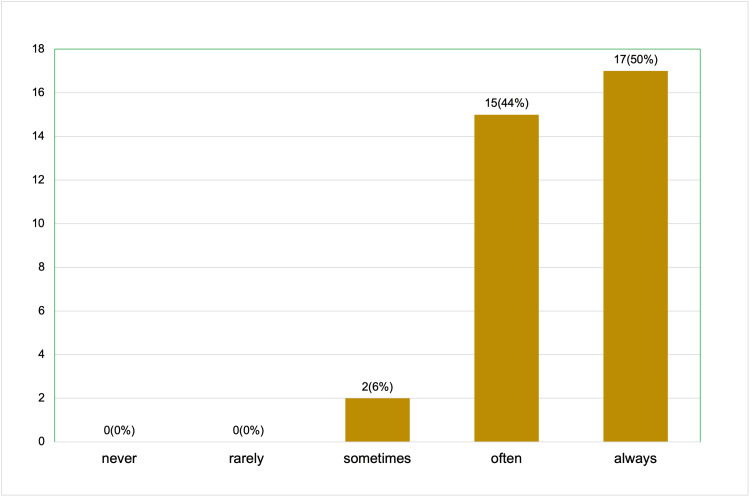
Post-session survey results (a total of 34 respondents): how often will you use the content from this presentation in your clinical practice?

Finally, when asked how often they would use the content from the presentation in their clinical practice, 17 participants (50%) indicated that they would always use it, and 15 (44%) reported they would often use it. Only 2 participants (6%) mentioned they would sometimes use the content, while no participants indicated they would rarely or never use it (Figure 6).

Survey responses were analyzed descriptively to compare pre- and post-session changes in participant familiarity, confidence, and self-reported use of TIC techniques. Frequencies and percentages were calculated for each Likert-style item using Microsoft Excel. No inferential statistical tests were performed, given the small sample size and the exploratory nature of this pilot study. All responses were treated anonymously, and only aggregate data were reported.

## Discussion

This workshop demonstrated measurable benefits in teaching TIC principles to EM learners, particularly in the context of performing sensitive physical examinations. The most notable shifts occurred in participants’ confidence and perceived preparedness to use trauma-informed language and physical exam maneuvers. Prior to the session, few learners reported confidence or regular use of these techniques. Following the workshop, however, the majority expressed strong confidence and a clear intent to apply the skills in their clinical practice.

Rather than simply improving knowledge, the session helped translate TIC concepts into actionable behaviors, an essential step toward reducing re-traumatization in acute care settings. Importantly, the interactive and case-based structure provided a psychologically safe space for reflection, deliberate practice, and skill-building, components that have been shown to improve retention and behavior change in adult learners.

The session was particularly relevant given the diverse and often vulnerable populations encountered in our ED, including refugees, immigrants, and survivors of GBV. As GBV continues to emerge as a pressing public health issue, EDs are frequently the first point of contact for survivors who may present with fear, apprehension, or mistrust. This training equipped EM clinicians with strategies to recognize signs of GBV and communicate with affected patients using empathy, safety, and empowerment as guiding principles. Such skills are critical for fostering trust, improving patient comfort, and facilitating timely identification and appropriate management of GBV-related concerns.

Beyond the learner experience, this initiative aligns with a growing recognition of the need to operationalize TIC in emergency settings. Existing literature underscores both the ethical imperative and practical feasibility of this work, particularly when TIC is framed as a universal precaution [16]. Embedding these approaches into EM training may help mitigate harm during high-stakes, time-pressured encounters where patient trust and safety are often fragile.

Our findings support the expansion of similar workshops across EM programs. Interdisciplinary teaching, especially with faculty experienced in trauma care and refugee health, strengthens realism and engagement. Future efforts should focus on developing scalable TIC curricula, building faculty development pathways, and assessing long-term effects on patient care and resident performance.

Limitations

This study has several limitations. The primary outcome, learner confidence, is self-reported and may not accurately reflect behavioral change or clinical competence. Although confidence is an important metric for educational interventions, it does not guarantee effective practice or improved patient experiences.

As a single-session pilot study, the findings should be interpreted cautiously. The descriptive design and absence of a control group, inferential statistics, or objective performance measures limit the ability to draw causal or generalizable conclusions. The use of Likert-style surveys introduces potential for response bias, including social desirability or overestimation of skill.

The single-institution sample also limits generalizability, particularly as participants were drawn from a residency program with existing institutional support for caring for refugee and trauma-affected populations. Furthermore, the short-term nature of the study restricts conclusions about sustained impact. Without longitudinal follow-up, it remains unclear whether participants retain or apply these skills months later or how this training influences real-world patient outcomes.

## Conclusions

This pilot study suggests that brief, focused TIC workshops are feasible and may enhance resident confidence and awareness when caring for patients affected by trauma, particularly refugees and immigrants. By providing practical strategies for conducting trauma-sensitive exams, the intervention demonstrated early promise in promoting compassionate, culturally responsive care within EM training. Future studies should incorporate objective performance assessments, such as simulated pelvic exams or delayed post-intervention surveys, to evaluate sustained behavioral change and the real-world impact of TIC education on clinical practice and patient experience.
